# A Novel SAVE Score to Stratify Decompensation Risk in Compensated Advanced Chronic Liver Disease (CHESS2102): An International Multicenter Cohort Study

**DOI:** 10.14309/ajg.0000000000001873

**Published:** 2022-06-15

**Authors:** Chuan Liu, Zhujun Cao, Huadong Yan, Yu Jun Wong, Qing Xie, Masashi Hirooka, Hirayuki Enomoto, Tae Hyung Kim, Amr Shaaban Hanafy, Yanna Liu, Yifei Huang, Xiaoguo Li, Ning Kang, Yohei Koizumi, Yoichi Hiasa, Takashi Nishimura, Hiroko Iijima, Young Kul Jung, Hyung Joon Yim, Ying Guo, Linpeng Zhang, Jianzhong Ma, Manoj Kumar, Ankur Jindal, Kok Ban Teh, Shiv Kumar Sarin, Xiaolong Qi

**Affiliations:** 1 CHESS Center, Center of Portal Hypertension, Department of Radiology, Zhongda Hospital, School of Medicine, Southeast University, Nanjing, China;; 2 Department of Infectious Disease, Ruijin Hospital, Shanghai Jiao Tong University School of Medicine, Shanghai, China;; 3 Department of Infectious Disease, Shulan Hospital, Hangzhou, China;; 4 Department of Gastroenterology & Hepatology, Changi General Hospital, SingHealth, Singapore;; 5 Duke-NUS Medical School, Singapore;; 6 Department of Gastroenterology and Metabology, Ehime University Graduate School of Medicine, Matsuyama, Japan;; 7 Department of Internal Medicine, Division of Gastroenterology and Hepatology, Hyogo College of Medicine, Nishinomiya, Japan;; 8 Division of Gastroenterology and Hepatology, Korea University Ansan Hospital, Ansan-si, Gyeonggi-do, Republic of Korea;; 9 Division of Gastroenterology, Hepatology and Endoscopy, Internal Medicine, Zagazig University Faculty of Medicine, Egypt;; 10 Ultrasound Imaging Center, Hyogo College of Medicine Hospital, Nishinomiya, Japan;; 11 Department of Hepatology, The Third People's Hospital of Taiyuan, Taiyuan, China;; 12 Department of Interventional Radiology, The Third People's Hospital of Taiyuan, Taiyuan, China;; 13 Department of Hepatology, Institute of Liver and Biliary Sciences (ILBS), New Delhi, India.

## Abstract

In patients with compensated advanced chronic liver disease (cACLD), the invasive measurement of hepatic venous pressure gradient is the best predictor of hepatic decompensation. This study aimed at developing an alternative risk prediction model to provide a decompensation risk assessment in cACLD.

**METHODS:**

Patients with cACLD were retrospectively included from 9 international centers within the Portal Hypertension Alliance in China (CHESS) network. Baseline variables from a Japanese cohort of 197 patients with cACLD were examined and fitted a Cox hazard regression model to develop a specific score for predicting hepatic decompensation. The novel score was validated in an external cohort (n = 770) from 5 centers in China, Singapore, Korea, and Egypt, and was further assessed for the ability of predicting clinically significant portal hypertension in a hepatic venous pressure gradient cohort (n = 285).

**RESULTS:**

In the derivation cohort, independent predictors of hepatic decompensation were identified including Stiffness of liver, Albumin, Varices, and platElets and fitted to develop the novel score, termed “SAVE” score. This score performed significantly better (all *P* < 0.05) than other assessed methods with a time-dependent receiver operating characteristic curve of 0.89 (95% confidence interval [CI]: 0.83–0.94) and 0.83 (95% CI: 0.73–0.92) in the derivation and validation cohorts, respectively. The decompensation risk was best stratified by the cutoff values at −6 and −4.5. The 5-year cumulative incidences of decompensation were 0%, 24.9%, and 69.0% in the low-risk, middle-risk, and high-risk groups, respectively (*P* < 0.001). The SAVE score also accurately predicted clinically significant portal hypertension (AUC, 0.85 95% CI: 0.80–0.90).

**DISCUSSION:**

The SAVE score can be readily incorporated into clinical practice to accurately predict the individual risk of hepatic decompensation in cACLD.

## INTRODUCTION

Once a decompensating event occurs, specifically ascites, variceal hemorrhage (VH), or hepatic encephalopathy, compensated advanced chronic liver disease (cACLD) or compensated cirrhosis becomes a systemic disease and life expectancy drops to ∼2 years (1). Portal hypertension is the main driver of hepatic decompensation (1,2). Currently, there are limited prognostic tools to predict the onset of decompensation in patients with cACLD.

Clinically significant portal hypertension (CSPH), defined as a hepatic venous pressure gradient (HVPG) of > 10 mm Hg, is the best validated prognostic tool in cACLD (1,2). Although HVPG is the gold standard to determine CSPH, it is invasive and resource-intensive, thus making it impractical for frequent follow-up (3). The presence of gastroesophageal varices (GEV) on endoscopy is another well-validated risk factor for decompensation, particularly VH (4). However, the mortality differs whether GEV presents as an isolated complication (20% 5-year mortality) or presents in association with other complications (over 80% 5-year mortality) (5). In a recent study of non-alcohol fatty liver disease (NAFLD)-related compensated cirrhosis, the presence of varices is associated with a 2-fold increase in decompensation risk but is increased to a ∼4- to 6-fold increase when combined with other markers of liver function and metabolism (6). It becomes clearer now that the routine surveillance of GEV in patients with cACLD is not sufficient and a predictive score for overall decompensation risk is urgently needed. The transient elastography for the liver stiffness measurement (LSM) has allowed the early identification of patients with chronic liver disease and is recommended to screen cACLD and CSPH by the Baveno VI consensus (1). LSM has previously been identified as a strong predictor of decompensation and death in patients with chronic liver disease (7). Data in cACLD are mainly derived from NAFLD population where baseline LSM and changes in LSM are independent risk factors of decompensation (8). Therefore, in addition to the surveillance of GEV, risk prediction modeling based on LSM could be an ideal tool for the triage of patients into those to be routinely followed up, those at risk of decompensation who would benefit from non-selective beta-blockers (NSBBs). This study aimed to develop and validate a novel ready-to-use score for decompensation risk stratification in an international multicenter cohort with complete baseline LSM, endoscopic screening, and clinical data.

## PATIENTS AND METHODS

### Study design

This international multicenter retrospective study was performed in cohorts from the Portal Hypertension Alliance in China (CHESS) network (study ID: CHESS2102). The aim of this study was to first explore the risk factors of the first hepatic decompensation in patients with cACLD. Our secondary aim was to develop and validate a ready-to-use score for risk stratification.

Risk factors were identified using the baseline (i.e., enrollment) data of 197 patients from Japanese cohorts as the derivation set. The novel score was then developed and used for risk stratification. External validation was performed in a multinational cohort with 770 patients from China, Singapore, Korea, and Egypt assessed between January 2009 and August 2020. Finally, the associations between the novel score, the severity of portal hypertension, and the presence of CSPH as assessed by HVPG were explored in 285 patients from China and India enrolled between July 2009 and August 2021.

All the data sets came from studies approved by ethical review boards of respective study sites. The informed consent for the medical information to be used for research was provided by patients or legal delegates from the participating centers. This study followed the Consolidated Standards of Reporting Trials and the Transparent Reporting of a multivariable prediction model for Individual Prognosis or Diagnosis guidelines for reports and was registered at the ClinicalTrials.gov (identifier: NCT04975477). All authors had access to the study data and had reviewed and approved the final article.

### Patients

Inclusion criteria were as follows: (i) adults aged 18 years or older, (ii) those who fulfilled cACLD diagnosis, and (iii) those who received endoscopic screening and LSM. The diagnosis of cACLD was made on (i) severe fibrosis or established cirrhosis on liver biopsy if available, (ii) GEV on endoscopy screening, (iii) HVPG > 5 mm Hg, or (iv) LSM ≥ 10 kpa according to the Baveno VI consensus (1).

The following exclusion criteria were applied: (i) prior hepatic decompensation, (ii) hepatocellular carcinoma, (iii) prior liver transplantation, (iv) portal vein thrombosis, (v) ongoing use of antiplatelet or anticoagulation, (vi) incomplete follow-up data, (vii) with NSBB treatment, and (viii) non-sinusoidal portal hypertension.

### Follow-up

All patients with cACLD were routinely followed up at 6-month intervals for the surveillance of hepatocellular carcinoma and decompensating events. Endoscopic surveillance was determined by the managing physician according to the recommended guidelines (1,9,10). As of August 13 2021 (the date of final data analysis), the median follow-up time was 50.1 (IQR: 34.3–65.6) months and 29.8 (IQR: 21.4–53.5) months in the derivation and validation cohorts, respectively.

### Main variables

Electronic medical records of all eligible subjects were reviewed to collect the following data at enrollment: demographics (age and sex), anthropometric variables (body mass index [BMI]), etiology of cirrhosis, routine laboratory data (alanine aminotransferase [ALT], aspartate aminotransferase [AST], total bilirubin [TB], creatinine [Cr] and international normalized ratio [INR], albumin, and platelet counts), LSM value by transient elastography, and the presence or absence of varices on endoscopic screening. With these variables, the following scores or criteria were applied: the Model for End-stage Liver Disease (MELD), ANTICIPATE model, ANTICIPATE non-alcoholic steatohepatitis (NASH), albumin-bilirubin (ALBI), ALBI-FIB-4, Baveno VII criteria for discerning CSPH (low risk: LSM≤15 kPa and platelet ≥150 × 10^9/L; high risk: LSM ≥ 25 kPa, middle risk: other), and Rete Sicilia Selezione Terapia–hepatitis C virus (RESIST-HCV) criteria for identifying patients without medium/large GEV (albumin > 36 g/L and platelet >120 × 10^9/L) (2,11–14). The etiology of cirrhosis was classified into 3 main categories including viral (hepatitis B, hepatitis C or both), NASH, and ALD, and the remaining etiologies including mixed ones were classified as other etiology.

### Measurement of LSM and HVPG

Liver stiffness of the patients from all participating centers was detected by using FibroScan (Echosens, Pairs, France) according to the manufacturer's instructions. The median value of successful measurements was taken to be the patient's LSM value and was expressed in kPa. The following criteria were used to define reliable LSM values: At least 10 valid measurements were obtained, interquartile range < 30% and successful rate > 60%. Patients with baseline unreliable LSM results were not included.

HVPG was performed in 3 centers (the Institute of Liver and Biliary Sciences in India, the Shulan Hospital of Hangzhou, and the Third People's Hospital of Taiyuan in China) by well-trained hepatologists or radiologists with a standard balloon-tipped catheter technique by experienced interventional specialists who were blinded to the patients' clinical data (2,15,16).

### Outcomes

The primary outcome of this study was the development of first hepatic decompensation at 1, 3, and 5 years, with death as the competing event. Patients were censored at the time of death and last follow-up, respectively. To minimize reporting bias in this retrospective study, we only included objective end points such as clinically significant ascites requiring diuretics, variceal bleeding documented by endoscopy, and hepatic encephalopathy defined as West-Haven grades 3–4 determined by specialists or requiring admission.

### Statistical analysis

Statistical analysis was performed using SPSS version 19.0 (IBM, Armonk, NY) and the timeROC package in R version 4.0.5 (R Foundation for Statistical Computing, Vienna, Austria). All statistical tests were 2-sided with a 5% significance level. Continuous variables and categorical variables were summarized and compared. Univariable and multivariable Cox proportional hazards regression models were used to estimate the effects of various variables on the hazard of decompensation and to develop the novel score. Forward likelihood ratio selection procedures were used for variable selection. Propensity score-matching (PSM) calculated by logistic regression based on baseline characteristics including TB, AST, ALT, albumin, platelet, varices, LSM, and etiology was applied to achieve a balance between derivation and validation cohorts. The time-dependent receiver operating characteristic curve (tAUC) was used to evaluate the prediction accuracy. Comparisons of accuracy were made with the deLong method between the novel score and other established scores. A restricted cubic spline was plotted to generate 2 optimal cutoff values to separate patients into the low-risk, middle-risk, and high-risk groups (17). Comparisons of decompensation probability curves among different risk groups were performed using the Gray test (18). Internal validation of the novel score was performed using the bootstrap method to assess the agreement between the probability of decompensation as predicted by the score and the observed probability. External validation was systemically performed in a multinational cohort with a large sample size (n = 770). Correlation between the score and the HVPG was performed using the Spearman rank test in an exploratory cohort. The diagnostic accuracy of the novel score for CSPH was assessed using AUC, sensitivity, specificity, positive predictive value, and negative predictive value.

## RESULTS

### Model derivation and validation cohorts

**Figure 1.**
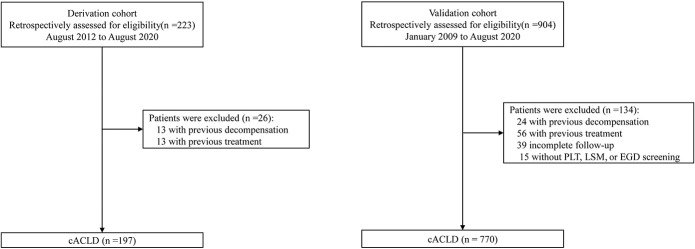
Flowchart of patient recruitment in the derivation and validation cohorts. cACLD, compensated advanced chronic liver disease; EGD, esophagogastroduodenoscopy; LSM, liver stiffness measurement; PLT, platelet.

Of all the 7 cohorts of cACLD with a long-term follow-up, 1,127 patients were assessed for eligibility and 967 patients were included in the score derivation and validation sets (see Figure 1 for patient recruitment diagram). Patients with cACLD in the derivation cohort were older, were a higher proportion of male subjects, had higher LSM, and had lower platelets and albumin than those in the validation cohort (*P* < 0.001). Consequently, the rates of varices were higher in the derivation cohort (Table 1). The etiology of cACLD in the derivation cohort was relatively balanced with one-third viral hepatitis-related, one-third NASH, 13.2% alcoholic, and 16.8% others. By contrast, the validation set consisted of predominantly viral hepatitis-related cACLD patients (83.2%) (Table 1).

**Table 1.**
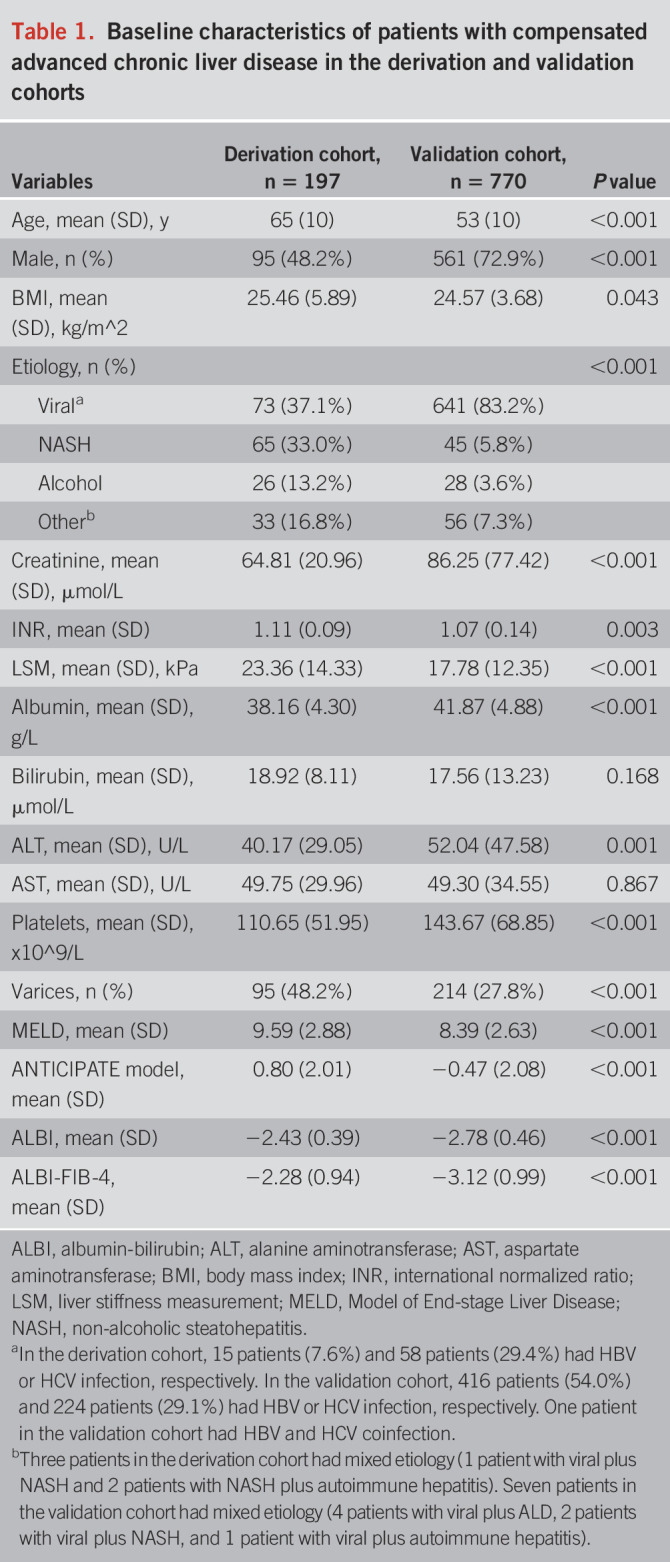
Baseline characteristics of patients with compensated advanced chronic liver disease in the derivation and validation cohorts

### Predictors of the first decompensation

During a median follow-up of 50.1 (interquartile range [IQR], 34.3–65.6) months, 53 patients (26.9%) developed an initial decompensation event in the derivation cohort. The cumulative incidences of decompensation were 2.6%, 16.7%, and 27.5% at 1, 3, and 5 years, respectively. In the univariable Cox regression analysis, LSM, platelets, varices, albumin, INR, and ALT were significantly associated with the onset of hepatic decompensation (Table 2).

**Table 2.**
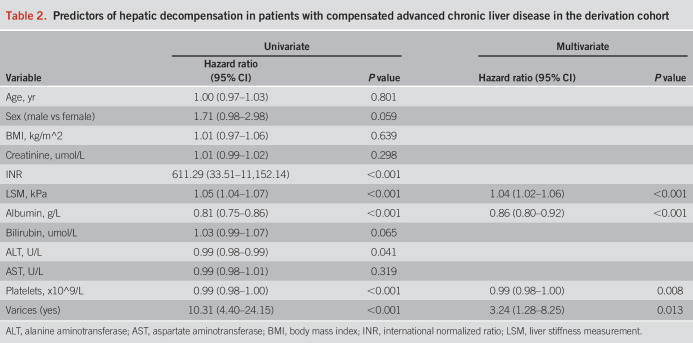
Predictors of hepatic decompensation in patients with compensated advanced chronic liver disease in the derivation cohort

### Derivation of the decompensation risk score

Multivariate Cox regression analysis further confirmed the presence of varices (hazard ratio [HR], 3.24; 95% confidence interval [CI], 1.28–8.25; *P* = 0.013), LSM (HR, 1.04; 95% CI, 1.02–1.06; *P* < 0.001), platelets (HR, 0.99; 95% CI, 0.98–1.00; *P* < 0.01), and albumin (HR, 0.86; 95% CI, 0.80–0.92; *P* < 0.001) as independent predictors of hepatic decompensation (Table 2). A novel risk score was then established based on Stiffness of liver, Albumin, Varices, and platElets and was named as the “SAVE” score (available as a free calculator at http://www.pan-chess.cn/calculator?modu=save_score):

SAVE score = 0.036* stiffness- 0.152* albumin- 0.011* platelets+ 1.177* [Varices: 0 if absent, 1 if present]

where liver stiffness is in kpa, albumin in g/L, and platelets in 10^9/L.

The novel score calibrated well with no significant differences between the observed and predicted probabilities of developing hepatic decompensation at 3 years in the derivation cohort (see Supplementary Figure 1a, Supplementary Digital Content 1, http://links.lww.com/AJG/C558).

### Discrimination ability of the SAVE score

**Figure 2.**
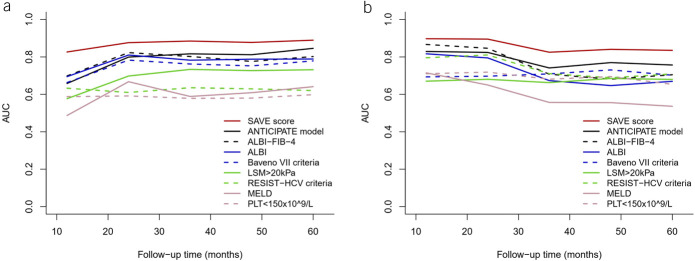
Summary time-dependent receiver operating characteristic curve for the Stiffness of liver, Albumin, Varices, and platElets (SAVE) score and other models to predict hepatic decompensation within the 5-year follow-up in the derivation (**a**) and validation (**b**) cohorts. ALBI, albumin‐bilirubin; MELD, Model of End‐stage Liver Disease; PLT, platelet; RESIST‐HCV, Rete Sicilia Selezione Terapia–hepatitis C virus.

The accuracy of the SAVE score in the derivation set to predict decompensating events at 3 years was significantly higher (tAUC 0.89, 95% CI, 0.83–0.94) than that of the ANTICIPATE model, ALBI, ALBI-FIB-4, Baveno VII criteria, RESIST-HCV criteria, or MELD scores (Table 3) and maintained with a tAUC of over 0.8 throughout the 5 years of the follow-up period (Figure 2a).

**Table 3.**
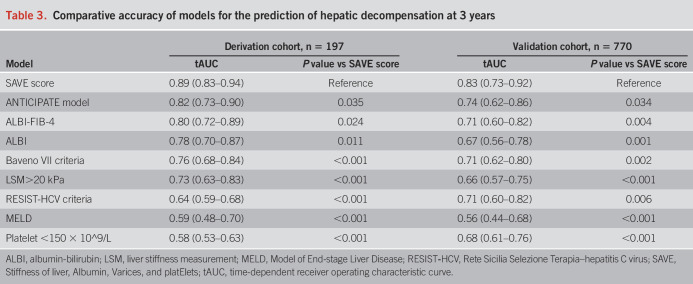
Comparative accuracy of models for the prediction of hepatic decompensation at 3 years

### Decompensation risk stratification based on the SAVE score

**Figure 3.**
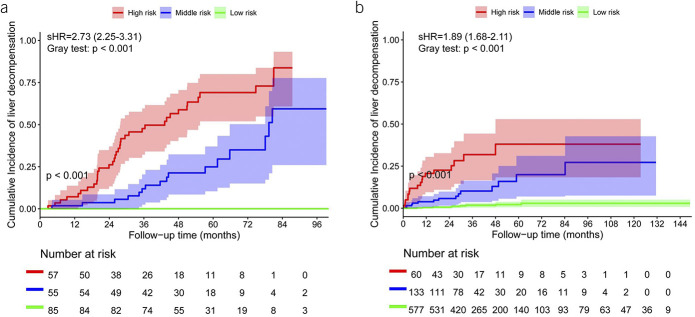
Cumulative incidence of first decompensation in patients with compensated advanced chronic liver disease stratified by the Stiffness of liver, Albumin, Varices, and platElets score in the derivation (**a**) and validation (**b**) cohorts. Cumulative incidence curves were calculated by competing risks regression taking death as a competing event. Comparison across different cumulative incidence curves was performed with the Gray test.

In the competing risk analysis (with death as a competing event), each point of the SAVE score would increase 2.73-fold (subdistribution hazard ratio [sHR], 95% CI 2.25-3.31, p < 0.001) risk of hepatic decompensation. A restricted cubic spline was then fitted and 2 nodes of the curve were selected as 2 optimal cutoff values (−6 and −4.5) to stratify the training cohort (n = 197) into low-risk (n = 85, 43.1%), middle-risk (n = 55, 27.9%), and high-risk (n = 57, 28.9%) groups, respectively (see Supplementary Figure 2, Supplementary Digital Content 1, http://links.lww.com/AJG/C558). The estimated cumulative incidences of decompensation at 1, 3, and 5 years were 0%, 0%, and 0% in the low-risk group vs 1.8%, 11.9%, and 24.9% in the middle-risk group and 7.2%, 47.7%, and 69.0% in the high-risk group, respectively (Gray test: *P* < 0.001) (Figure 3a).

### Validation of the SAVE score

The SAVE score worked well in the validation cohort with good calibration (see Supplementary Figure 1b, Supplementary Digital Content 1, http://links.lww.com/AJG/C558), and the risk of developing decompensation progressively increased with the increase in the SAVE score (sHR, 1.89 95% CI 1.68–2.11, Gray test *P* < 0.001). It performed significantly better than the ANTICIPATE model, ALBI, ALBI-FIB-4, Baveno VII criteria, RESIST-HCV criteria, or MELD scores (Figure 2b) with a tAUC of 0.83 (0.73–0.92) in predicting 3-year decompensation (Table 3).

A total of 577 (74.9%), 133 (17.2%), and 60 (7.8%) patients in the validation cohort were assigned to the low-risk, middle-risk, and high-risk groups, respectively, according to the SAVE score. Comparing with the low-risk group, the middle-risk and high-risk groups had an 8.02-fold (sHR, 95% CI, 3.46–18.55, *P* < 0.001) and 24.53-fold (sHR, 95% CI, 10.90–55.17, *P* < 0.001) higher risk of developing hepatic decompensation. The estimated cumulative incidences of hepatic decompensation at 1, 3, and 5 years were 0.4%, 1.7%, and 2.2% in the low-risk group vs 3.9%, 10.2%, and 19.9% in the middle-risk group and 20.7%, 31.8%, and 38.0% in the high-risk group, respectively (Gray test: *P* < 0.001) (Figure 3b).

In subgroup analysis, the SAVE model performed well in different etiology groups with 3-year tAUC ≥ 0.8 (viral: 3-year tAUC 0.81 [0.70–0.93]; NASH: 3-year tAUC 0.89 [0.76–1.00]; ALD: 3-year tAUC 0.80 [0.56–1.00]; other etiology: 3-year tAUC 0.82 [0.57–1.00]). In the NASH group, 3-year tAUC of the SAVE model was higher than ANTICIPATE NASH (3-year tAUC 0.74 [0.54–0.94]).

### Sensitivity analysis

PSM analysis was performed to achieve a more comparable validation set to the derivation cohort. In the PSM matched cohorts, LSM, platelets, albumin, and rate of varices were balanced (see Supplementary Table 1, Supplementary Digital Content 2, http://links.lww.com/AJG/C559). The 3-year tAUC of the SAVE score was 0.83 (0.73–0.93) in the matched validation cohort, higher than other models (all *P* < 0.05, Table 4, see Supplementary Figure 3, Supplementary Digital Content 1, http://links.lww.com/AJG/C558). Similar to the results observed in the prematched cohorts, the cumulative incidence of decompensation elevated in parallel with the increase of the SAVE score.

**Table 4.**
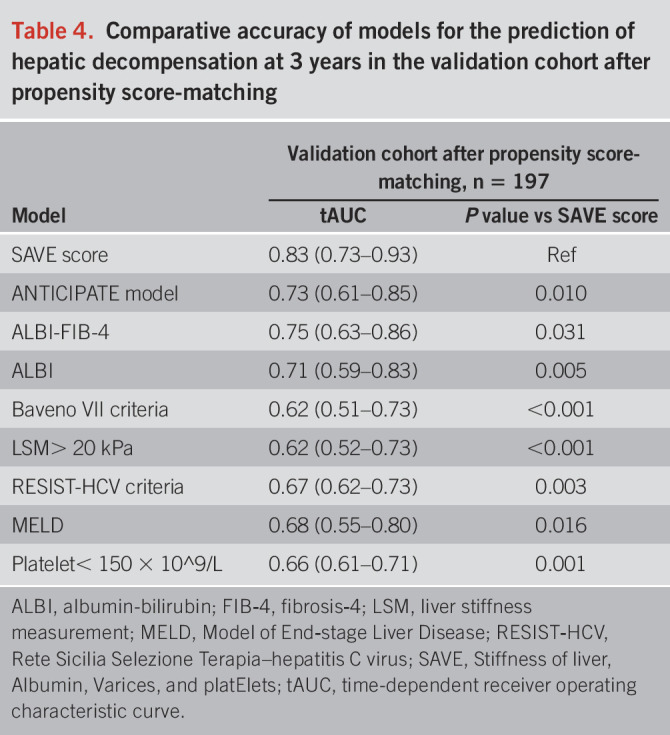
Comparative accuracy of models for the prediction of hepatic decompensation at 3 years in the validation cohort after propensity score-matching

### Exploratory analysis

**Figure 4.**
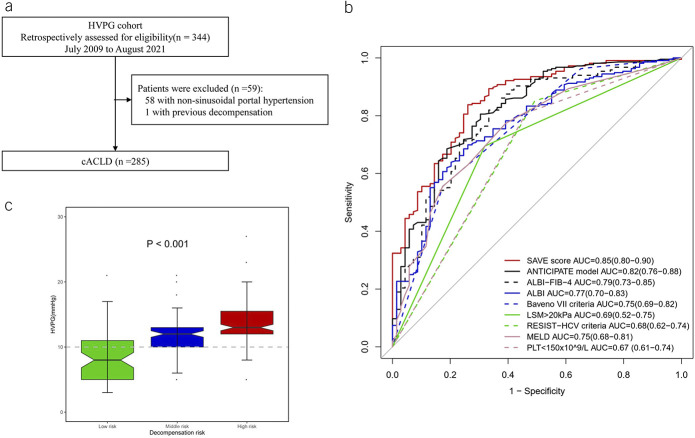
Exploratory analysis of the SAVE score in association with hepatic venous pressure gradient and prediction of clinically significant portal hypertension. (**a**) Flowchart of patient recruitment in the HVPG cohort, (**b**) comparisons of the SAVE score with other methods in predicting the presence of clinically significant portal hypertension, and (**c**) the distribution of HVPG in low‐risk, middle-risk, and high‐risk groups, respectively. ALBI, albumin‐bilirubin; FIB‐4, fibrosis; MELD, Model of End‐stage Liver Disease; PLT, platelet; RESIST‐HCV, Rete Sicilia Selezione Terapia–hepatitis C virus; SAVE, Stiffness of liver, Albumin, Varices, and platElets.

Based on the good accuracy of the SAVE score in predicting portal hypertension-related decompensating events, we further explored the association between the HVPG and the SAVE score. The characteristics of the cohort (n = 285) (Figure 4a) used for this analysis are summarized in Supplementary Table 2, Supplementary Digital Content 2, http://links.lww.com/AJG/C559. The AUC of the SAVE in diagnosing CSPH was 0.85 (95% CI: 0.80–0.90, *P* < 0.05), significantly higher than that of other models except for the ANTICIPATE model (Figure 4b). In patients with NASH of the HVPG cohort, the AUC of the SAVE score was 0.83 (0.74–0.92), higher than ANTICIPATE NASH with 0.80 (95% CI: 0.69–0.91). In line with the results from the prognostic analysis using the SAVE score as a risk-stratifying tool, a −6 point of the SAVE score was highly sensitive to rule out CSPH with a sensitivity of 0.90 (95% CI: 0.86–0.94) and a −4.5 point of the SAVE was highly specific to rule in CSPH with a PPV of 0.94 (95% CI: 0.91–0.98) (Figure 4b). The median HVPG of low-risk, middle-risk, and high-risk groups were 8.00 (5.00–11.00), 12.00 (10.00–13.50), and 13.00 (12.00–16.00), respectively (Figure 4c).

## DISCUSSION

The development of ascites, VH, or HE marks the transition from cACLD to the decompensated stage. With the onset of decompensation, the median survival dramatically falls from ∼12 years at the compensated stage to less than 2 years (19,20). The annual risk of decompensation varies with disease etiology, ranging from 4% to more than 10% (21) and is attributed but not limited to the progression of portal hypertension. There is a clear unmet need to develop an accurate score for the early identification of patients with cACLD at high risk of decompensation.

In this study, we developed a novel risk stratification tool, namely, SAVE score, in an etiology balanced cohort and externally validated the score in an international cohort with a large sample size. Our findings showed that the SAVE score (including stiffness, albumin, varices, and platelet) had excellent predictive accuracy for hepatic decompensation at both 3-year and 5-year time points. The novel score calibrated well and was more accurate in comparison with other existing noninvasive fibrosis scores and prognostic models in cACLD. These results were further validated in the PSM analysis where baseline differences between derivation and validation cohorts were controlled. The SAVE score calculator is now free available at http://www.pan-chess.cn/calculator?modu=save_score established by the CHESS consortium.

Portal hypertension is the main pathophysiologic driver of the initial decompensating events. Therefore, it is not surprising that 3 of the 4 components in the SAVE score are highly related to the severity of portal hypertension. Liver stiffness is a strong and validated predictor of the first decompensation in patients with cACLD. In previous studies, its accuracy was similar to that of HVPG for predicting decompensation (7,22). Together with platelet count > 150,000, a liver stiffness of < 20 kPa has also been recommended by the Baveno VI consensus to spare unnecessary endoscopies due to a very low risk of having varices requiring treatment (1). The presence of GEV on endoscopy is a hallmark of portal hypertension and is associated with an annual rate of around 10%–15% developing VH. The overall 6-year incidence of hepatic decompensation significantly increased from 26% to 66% once GEV is present in HCV cirrhosis (23). In a recent study of NAFLD-related cirrhosis, the presence of GEV increases the risk of decompensation by 2-folds (6). Similarly, we found that the presence of GEV was associated with a 3.2-fold increase in the risk of decompensation in our cohort of patients with all etiology of cirrhosis. By performing the exploratory analysis, we further validated the strong association between the SAVE score and the severity of portal hypertension by showing a significant positive correlation of the SAVE score with the HVPG value.

It is also important to recognize that liver insufficiency also plays an important role in the development of decompensation. Serum albumin has been identified as an independent risk factor for developing decompensation in our study. This is consistent with the previous study (24). Albumin is a marker of liver synthetic function and is a major regulator of body fluid distribution (oncotic property). In addition to portal hypertension, hypoalbuminemia is another pathophysiologic driver of ascites. Recently, the understanding of the albumin function in patients with cirrhosis has expanded to its non-oncotic properties including antioxidant property, immune modulation, and its capacity of binding and transportation of many endogenous and exogenous substances, thereby contributing to the maintenance of the normal capillary permeability (19). The ANSWER study has demonstrated that long-term administration of human albumin in patients with cirrhosis and ascites reduces the probability of developing ascites and hospital readmissions (25). Our study again highlighted the risk of developing future complications in patients with compensated cirrhosis and hypoalbuminemia. Further studies are needed to address whether the administration of human albumin in such a population would reduce the incidence of decompensation or delay it.

Moving from pathophysiology to the clinical ground, it should be highlighted that the SAVE score is based on the results of the endoscopic screening. However, with the addition of other parameters, the application of the SAVE score into clinical practice would help us shift the existing paradigm (find and treat those with high-risk varices to prevent VH) to a new paradigm (find and treat those at high-risk decompensation to prevent any decompensating event) (26). The PREDESCI study has shown that the use of NSBBs in patients with CSPH reduces the incidence of decompensation and increases decompensation-free survival (27). Therefore, the SAVE score would be a useful tool to guide the use of NSBBs once cACLD is diagnosed. For example, patients with middle or high risk may benefit from NSBBs because they are very likely to have CSPH with a 20% or 60% probability of developing decompensation at 5 years. However, it should be noted that with the increasing recommendation of carvedilol from the updated Baveno VII guidelines, the need for endoscopy is moving from the mainstream. Scores free of endoscopy are needed to be developed for the prediction of decompensation in cirrhosis in the future.

Our study also has a few limitations. First, in this international retrospective multicenter study, we cannot control patients' enrollment and management flow through a prespecified study protocol. To minimize the impact, we use strict inclusion and exclusion criteria to pursue a representative study population. Laboratory tests were also performed in site laboratories; however, to assure the comparability of the laboratory results, each variable was transformed into the same units and normal ranges. We also like to acknowledge that serum albumin is a negative phase reactant and could be confounded in patients with nephrotic syndrome. Second, the performance of the SAVE score was well validated in a population with mainly Asians and virus-related cirrhosis, but patients of other ethnicities (e.g., White and African) and with other etiologies, particularly NASH, require further investigation from other regions. Third, the follow-up time of the cohorts was not long enough to analyze the predictive performance of the SAVE score at 10 years. Future studies are warranted to address this point. Finally, the requirement of endoscopy and elastography decreases the applicability of the SAVE score in general clinical settings. Scores based on routinely available laboratory variables warrant further investigations.

In conclusion, the SAVE score, a combination of laboratory, imaging, and endoscopic assessment, optimizes the prediction of hepatic decompensation. This is a ready-to-use clinical tool to tailor monitoring and treatment strategies in patients with cACLD.

## CONFLICTS OF INTEREST

**Guarantor of the article:** Xiaolong Qi, MD, PhD.

**Specific author contributions**: Study conception: X.Q. and C.L. Data acquisition: C.L., Z.C., H.Y., Y.J.W., Q.X., M.H., H.E., T.H.K., A.S.H., Y.L., Y.H., X.L., N.K., Y.K., Y.H., T.N., H.I., Y.K.J., H.J.Y., Y.G., L.Z., J.M., M.K., A.J., K.B.T., S.K.S., and X.Q.. Statistical analysis: C.L. and Z.C. Drafting the initial manuscript: C.L., Z.C., and X.Q. Critical review of the manuscript: X.Q. and Y.J.W. All authors reviewed and approved the final version of the manuscript.

**Financial support:** National Natural Science Foundation of China (No. 82000588) and Shanghai Municipal Key Clinical Specialty (shslczdzk01103). The funding sources were not involved in study design, data collection, analysis and interpretation of the data, or writing of the report or decision to submit for publication.

**Potential competing interests:** None to report.

**Study Highlights**

**WHAT IS KNOWN**

✓ The annual risk of hepatic decompensation varies with disease etiology from 4% to more than 10% in patients with compensated advanced chronic liver disease (cACLD).
✓ The severity of portal hypertension assessed through the invasive measurement of hepatic venous pressure gradient (HVPG) is the best validated predictor of decompensation in cACLD.
✓ Endoscopic surveillance for varices and liver stiffness measured by transient elastography are suboptimal alternatives to HVPG for the prediction of decompensation.

**WHAT IS NEW HERE**

✓ In an international collaboration involving 1,252 patients with cACLD, a novel score incorporating Stiffness of liver, Albumin, Varices, and platElets (SAVE) predicts hepatic decompensation better than albumin-bilirubin, albumin-bilirubin-FIB-4, ANTICIPATE model, Baveno VII criteria, Rete Sicilia Selezione Terapia–hepatitis C virus criteria, and Model for End-stage Liver Disease scores.
✓ The SAVE score stratified patients with cACLD into low-risk, middle-risk, and high-risk groups with a stepwise increase of 3- and 5-year decompensation rates.
✓ The SAVE score correlated well with the HVPG value and accurately predicted the presence of clinically significant portal hypertension.

## Supplementary Material
