# Supplementary material for: A Novel SAVE Score to Stratify Decompensation Risk in Compensated Advanced Chronic Liver Disease (CHESS2102): An International Multicenter Cohort Study
Source: Am J Gastroenterol. 2022 Jun 15;117(10):1605–13. doi: 10.14309/ajg.0000000000001873 (PMC9531993; doi:10.14309/ajg.0000000000001873)
Supplement: Supplementary file 1 [file acg-117-1605-s001.pdf]

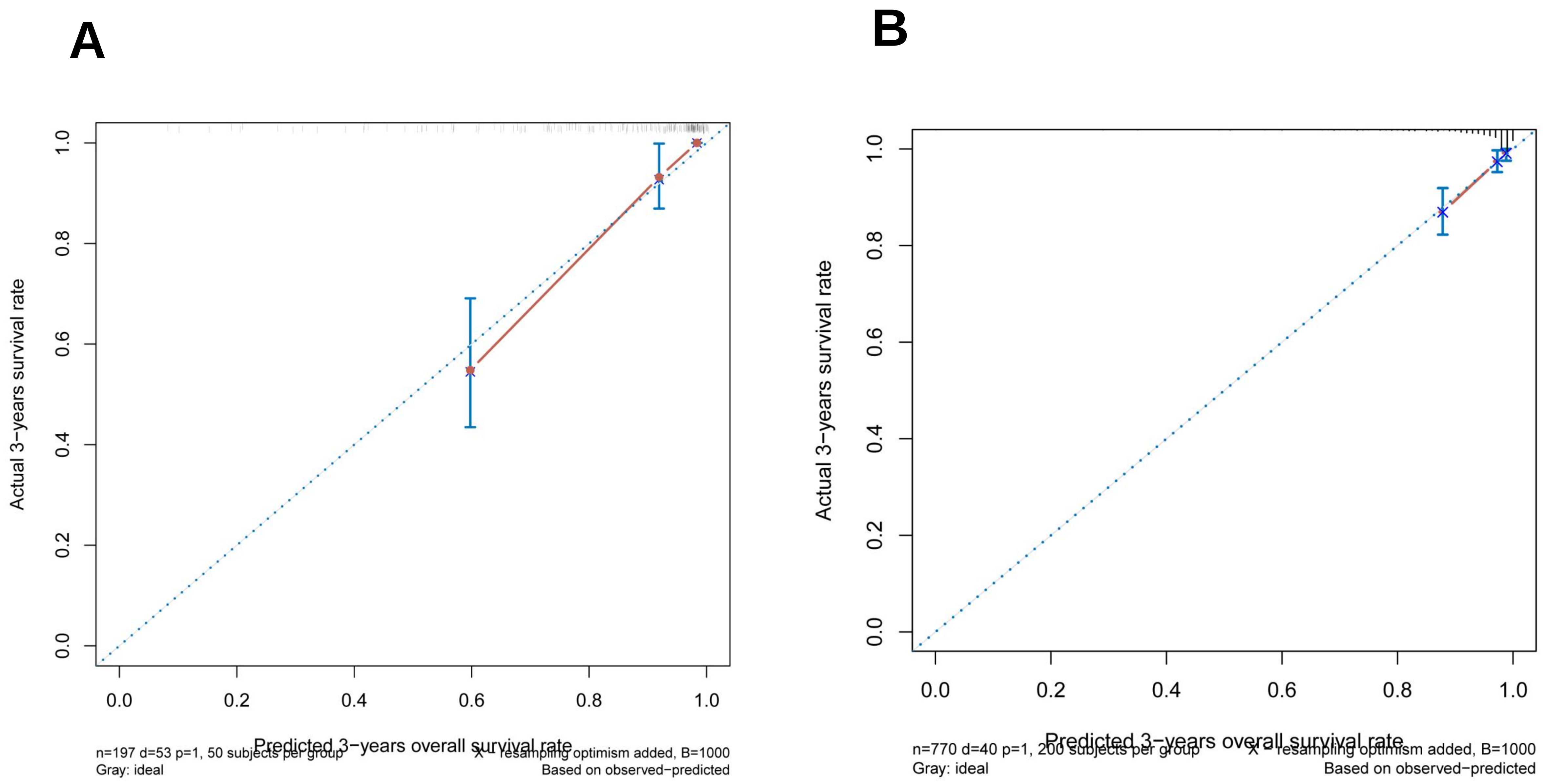

Supplementary Figure 1: Calibration curves of the SAVE score to predict decompensation in the derivation (A) and the validation (B) cohort.

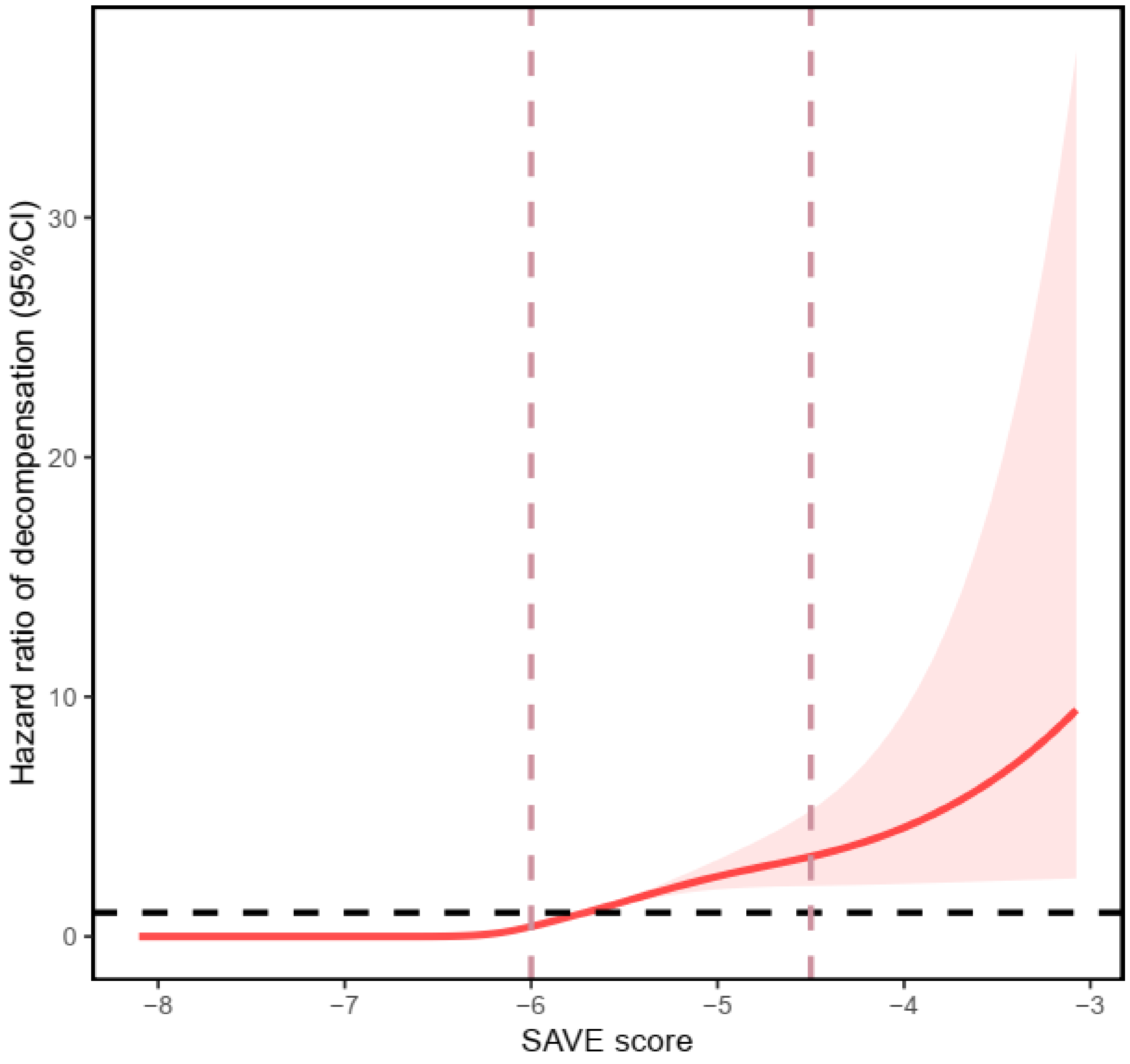

Supplementary Figure 2: Restricted cubic spline of the SAVE score fitted with Cox-hazard regression and two nodes of the curve were selected as 2 optimal cut-off values (-6 and -4.5) to stratify patients into low risk (SAVE score <-6), middle risk (-6 to -4.5) and high risk (SAVE score >-4.5).

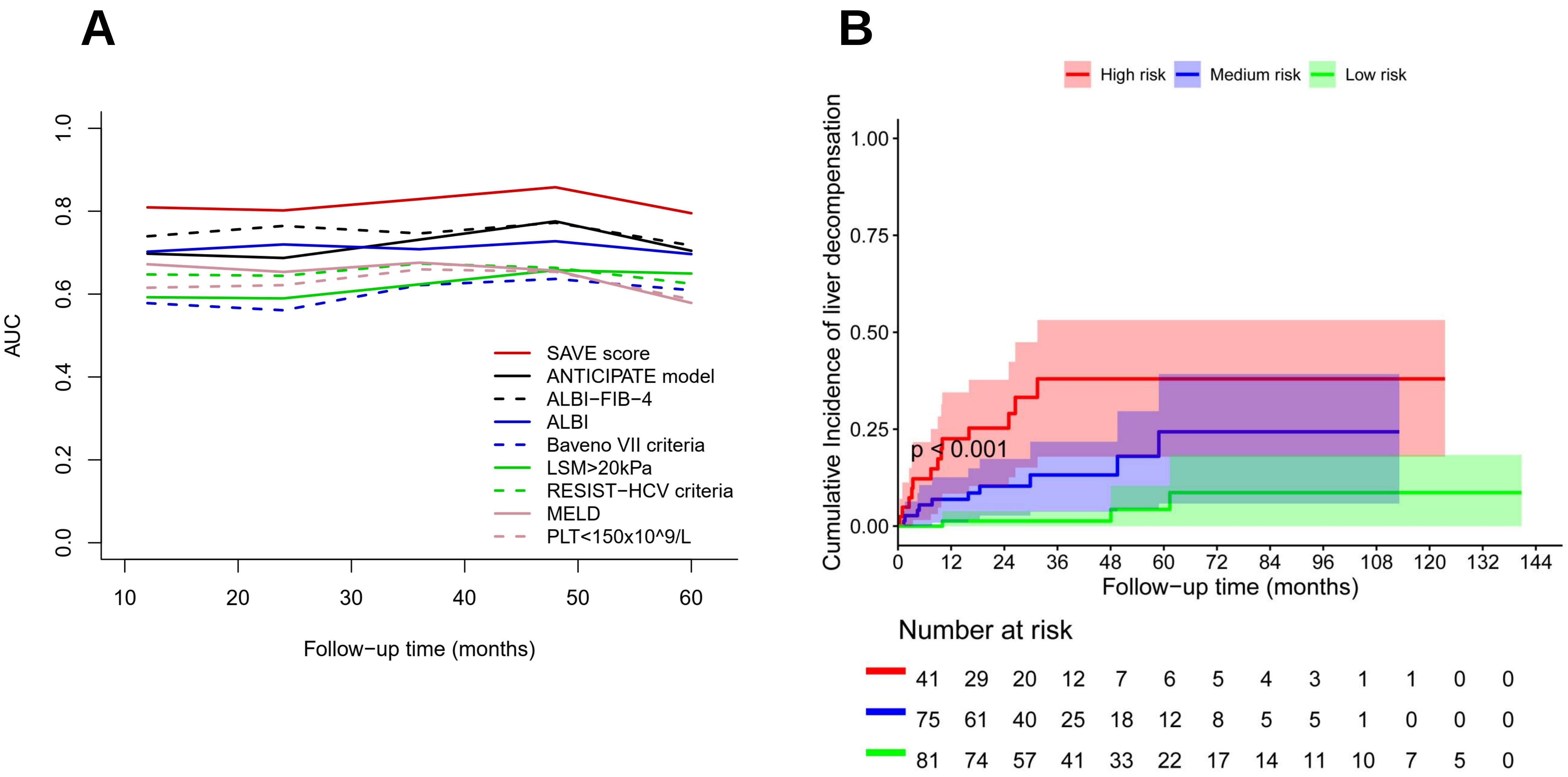

Supplementary Figure 3: The performance of SAVE score for predicting hepatic decompensation in Validation cohort after PSM. Summary tAUCs for the SAVE score and other methods to predict hepatic decompensation (A) and cumulative incidence of first decompensation in patients with cACLD stratified by SAVE score (B).
