# Supplementary material for: A Novel SAVE Score to Stratify Decompensation Risk in Compensated Advanced Chronic Liver Disease (CHESS2102): An International Multicenter Cohort Study
Source: Am J Gastroenterol. 2022 Jun 15;117(10):1605–13. doi: 10.14309/ajg.0000000000001873 (PMC9531993; doi:10.14309/ajg.0000000000001873)
Supplement: Supplementary file 2 [file acg-117-1605-s002.docx]

**Supplementary Table 1 Baseline characteristics of patients with cACLD in validation cohorts after propensity score matching**

| Variables | Validation cohort after propensity score matching  n= 197 | p value vs Derivation cohort |
| --- | --- | --- |
| Age, mean (SD), y | 56(11) | <0.001 |
| Male, n (%) | 112(56.9%) | 0.086 |
| BMI, mean (SD), kg/m^2 | 25.08(4.11) | 0.458 |
| Etiology, n (%) |  | <0.001 |
| Viral^#^ | 109(55.3%) |  |
| NASH | 26(13.2%) |  |
| Alcohol | 12(6.1%) |  |
| Other^&^ | 50(25.4%) |  |
| Creatinine, mean (SD), umol/L | 85.48(87.96) | 0.002 |
| INR, mean (SD) | 1.12(0.16) | 0.421 |
| LSM, mean (SD), kPa | 23.61(15.97) | 0.869 |
| Albumin, mean (SD), g/L | 38.17(5.77) | 0.980 |
| Bilirubin, mean (SD), umol/L | 19.69(17.86) | 0.583 |
| ALT, mean (SD), U/L | 39.78(28.72) | 0.892 |
| AST, mean (SD), U/L | 52.07(35.64) | 0.485 |
| Platelets, mean (SD), x10^9/L | 111.06(53.05) | 0.938 |
| Varices, n (%) | 98(49.7%) | 0.762 |
| MELD, mean (SD) | 9.02(3.20) | 0.062 |
| ANTICIPATE model | 0.77(2.08) | 0.874 |
| ALBI, mean (SD) | -2.44(0.55) | 0.828 |
| ALBI-FIB-4, mean (SD) | -2.33(1.32) | 0.670 |

*Abbreviations:*

ALT= alanine aminotransferase, AST= aspartate aminotransferase, INR= international normalized ratio, LSM= liver stiffness measurement, NASH= non-alcoholic steatohepatitis, MELD= model of end stage liver disease, SD= standard difference

# Seventy-six patients (38.5%) and 33 patients (16.7%) had HBV or HCV infection, respectively.

& Five patients had mixed etiology (2 patients with viral plus ALD; 2 patients with viral plus NASH; 1 patient with viral plus AIH).

**Supplementary Table 2:** **Baseline characteristics of the HVPG cohort**

| Variable | HVPG cohort  n= 285 |
| --- | --- |
| Age, mean (SD), y | 53(10) |
| Gender, n (%) | 217(76.1%) |
| Creatinine, mean (SD), umol/L | 79.84(67.61) |
| INR, mean (SD) | 1.27(0.28) |
| LSM, mean (SD), kPa | 24.49(13.80) |
| Albumin, mean (SD), g/L | 34.33(6.78) |
| Bilirubin, mean (SD), umol/L | 33.67(43.81) |
| ALT, mean (SD), U/L | 57.58(97.11) |
| AST, mean (SD), U/L | 73.51(119.53) |
| Platelets, mean (SD), x10^9/L | 128.65(64.98) |
| Varices, n (%) | 217(76.1) |
| MELD, mean (SD) | 11.17(4.24) |
| HVPG, mean (SD), mmHg | 11.78(3.74) |
| Etiology, n (%) |  |
| Viral^#^ | 133(46.7%) |
| NASH | 91(31.9%) |
| Alcohol | 61(21.4%) |

*Abbreviations:*

ALT= alanine aminotransferase, AST= aspartate aminotransferase, INR= international normalized ratio, LSM= liver stiffness measurement, NASH= non-alcoholic steatohepatitis, MELD= model of end stage liver disease, SD= standard difference, HVPG= hepatic venous pressure gradient

# Seventy-one patients (24.9%) and 62 patients (21.8%) had HBV or HCV infection, respectively.
